# Neuroinflammation and Alzheimer’s Disease: A Machine Learning Approach to CSF Proteomics

**DOI:** 10.3390/cells10081930

**Published:** 2021-07-29

**Authors:** Lorenzo Gaetani, Giovanni Bellomo, Lucilla Parnetti, Kaj Blennow, Henrik Zetterberg, Massimiliano Di Filippo

**Affiliations:** 1 Section of Neurology, Department of Medicine and Surgery, University of Perugia, 06100 Perugia, Italy; lucilla.parnetti@unipg.it; 2 Laboratory of Clinical Neurochemistry, Department of Medicine and Surgery, University of Perugia, 06100 Perugia, Italy; giovanni.bellomo@unipg.it; 3 Institute of Neuroscience and Physiology, Department of Psychiatry and Neurochemistry, The Sahlgrenska Academy at the University of Gothenburg, 431 41 Mölndal, Sweden; kaj.blennow@neuro.gu.se (K.B.); henrik.zetterberg@clinchem.gu.se (H.Z.); 4 Clinical Neurochemistry Laboratory, Sahlgrenska University Hospital, 431 41 Mölndal, Sweden; 5 Department of Neurodegenerative Disease, UCL Institute of Neurology, Queen Square, London WC1N 3BG, UK; 6 UK Dementia Research Institute at UCL, London WC1E 6B, UK

**Keywords:** Alzheimer’s disease, CSF biomarkers, proximity extension assay, neuroinflammation, SIRT2, HGF, MMP-10, CXCL5

## Abstract

In Alzheimer’s disease (AD), the contribution of pathophysiological mechanisms other than amyloidosis and tauopathy is now widely recognized, although not clearly quantifiable by means of fluid biomarkers. We aimed to identify quantifiable protein biomarkers reflecting neuroinflammation in AD using multiplex proximity extension assay (PEA) testing. Cerebrospinal fluid (CSF) samples from patients with mild cognitive impairment due to AD (AD-MCI) and from controls, i.e., patients with other neurological diseases (OND), were analyzed with the Olink Inflammation PEA biomarker panel. A machine-learning approach was then used to identify biomarkers discriminating AD-MCI (*n*: 34) from OND (*n*: 25). On univariate analysis, SIRT2, HGF, MMP-10, and CXCL5 showed high discriminatory performance (AUC 0.809, *p* = 5.2 × 10^−4^, AUC 0.802, *p* = 6.4 × 10^−4^, AUC 0.793, *p* = 3.2 × 10^−3^, AUC 0.761, *p* = 2.3 × 10^−3^, respectively), with higher CSF levels in AD-MCI patients as compared to controls. These same proteins were the best contributors to the penalized logistic regression model discriminating AD-MCI from controls (AUC of the model 0.906, *p* = 2.97 × 10^−7^). The biological processes regulated by these proteins include astrocyte and microglia activation, amyloid, and tau misfolding modulation, and blood-brain barrier dysfunction. Using a high-throughput multiplex CSF analysis coupled with a machine-learning statistical approach, we identified novel biomarkers reflecting neuroinflammation in AD. Studies confirming these results by means of different assays are needed to validate PEA as a multiplex technique for CSF analysis and biomarker discovery in the field of neurological diseases.

## 1. Introduction

Alzheimer’s disease (AD) is the commonest cause of dementia in the elderly, and the most frequent human neurodegenerative disease worldwide [1]. In AD, amyloid-β (Aβ) pathology has long been considered as the central event in the pathophysiology of the disease, followed by the intraneuronal aggregation of misfolded and phosphorylated tau protein in form of neuritic plaques, neurofibrillary tangles, and neuropil threads, leading to neurodegeneration and ultimately neuronal death [2].

However, evidence suggests that amyloid and tau pathologies alone cannot explain AD pathophysiology. In this context, both the discovery of elevated levels of inflammatory markers in AD and of AD risk genes associated with innate immune function, suggest a potential contribution of neuroinflammation in AD pathogenesis [3]. Neuroinflammation refers to an abnormal inflammatory response within the central nervous system (CNS) that can be induced by a variety of triggers (e.g., infective, traumatic, vascular, or toxic insults) [4]. A pivotal role in neuroinflammation is played by CNS innate immune system cells, mainly microglia, together with astrocytes, endothelial cells responsible for blood-brain barrier (BBB) dysfunction, and infiltrating peripheral white blood cells [5].

Studies performed on animal models of neurodegeneration, as well as histological findings from AD brains, in vivo studies performed using brain positron emission tomography (PET) and, finally, evidence from human genetic diseases, have highlighted the possible contribution of neuroinflammation in AD [6]. However, whether neuroinflammation is a driving pathophysiological mechanism rather than a downstream effect of neurodegeneration is still a matter of debate [7].

The cerebrospinal fluid (CSF) has been extensively investigated in AD as a source of biomarkers, and a variety of studies has been performed on the levels of inflammatory and glial markers in AD patients compared with controls [6]. Multiplex analyses have also been performed on a more accessible matrix, such as plasma [8]. However, results have not been homogeneous between studies, measured biomarkers were restricted to a small number of inflammatory proteins, and the applied multiplex analyses were limited by antibody cross-reactivity and inter-assay variability. Recently, multiplex mass-spectrometry analyses have been applied to both CSF and brain tissues in AD, confirming the potential contribution of glial markers [9].

Proximity extension assay (PEA) technology is a 96-plex immunoassay for fluid protein detection, which relies on unique antibody–oligonucleotide protein binding for quantitative measurement by means of real-time polymerase chain reaction (PCR) [10]. PEA technology is less exposed to the risk of multiplex analyses limitations, and it has been previously applied to the CSF in neurological disorders, namely Parkinson’s disease and atypical parkinsonian syndromes [11], as well as in the blood in AD patients [12].

Since multiplex analyses provide many outcome variables, their interpretation may be non-trivial and there is a high risk of data overfitting. Machine learning approaches based on penalized regression analysis may therefore allow identifying the smallest possible panel of novel markers with high sensitivity and specificity.

Herein, we have used PEA technology to measure a broad panel of biomarkers reflecting immune activation in CSF samples from patients with early-stage AD and from a control group. To identify the proteins that best separated the AD and control groups, we applied machine-learning statistical models, the results of which may offer a significant contribution to biomarker discovery and novel insights into the pathophysiology of AD.

## 2. Patients and Methods

### 2.1. Patients Selection

A retrospective cohort of patients with mild cognitive impairment due to AD (AD-MCI) and other neurological diseases (OND) as the control group, with CSF samples stored in the Laboratory of Clinical Neurochemistry, Department of Medicine and Surgery, University of Perugia (Perugia, Italy), was selected for this study.

AD-MCI patients were enrolled among individuals referring to the Center for Memory Disturbances of the University Hospital of Perugia who underwent lumbar puncture as part of the routine diagnostic workup between 2008 and 2016. Beyond lumbar puncture, all patients underwent a clinical neurological examination, neuropsychological assessment including a Mini-Mental State Examination (MMSE) and Clinical Dementia Rating (CDR) scale, blood chemistry, brain computed tomography (CT), and/or magnetic resonance imaging (MRI) scan for ruling out differential diagnoses. All the patients underwent a clinical follow-up of at least 1 year. Patients with a CSF biomarker profile suggestive of amyloidosis (A+) and tauopathy (T+), were selected for the study [13].

Patients in the OND group were selected among individuals undergoing lumbar puncture for diagnostic purposes other than cognitive complaints and for whom the eventual diagnosis did not include inflammatory or degenerative neurological diseases. For this cohort of control subjects, CSF biomarker profile was not suggestive of amyloidosis (A-), tauopathy (T-), and neurodegeneration (N-).

All the patients were 55–85 years old and none of them had undergone steroid or other immunosuppressant/immunomodulatory treatments within 30 days before lumbar puncture. The local ethics committee approved the study (Protocol N°: 19369/08/AV, registry N°: 1287/08).

### 2.2. CSF Collection and Storage

CSF samples were collected over an 8-year period (2008–2016) in the Section of Neurology, Department of Medicine and Surgery, University of Perugia (Perugia, Italy). CSF samples were collected and stored with standardized procedures. Specifically, samples were obtained by means of lumbar puncture, performed between 8:00 a.m. and 11:00 a.m. CSF samples were collected in sterile polypropylene tubes, centrifuged for 10 min at 2000× *g*, divided into 0.5 mL aliquots, and immediately frozen at −80 °C, pending analysis. Collection and storage of CSF samples were carried out by following specific international guidelines [14].

### 2.3. CSF ATN Profile

In the CSF samples collected for the study, Aβ42, t-tau, and p-tau levels were previously measured by using INNOTEST ELISA (Fujirebio Europe, Gent, Belgium) at the Laboratory of Clinical Neurochemistry, Department of Medicine and Surgery, University of Perugia (Perugia, Italy). Depending on the lumbar puncture date, CSF samples were classified as A+ for Aβ42 ≤ 800 (2011–2016) or ≤ 1200 (2008–2010) pg/mL, T+ for p-tau ≥ 60 pg/mL and N+ for t-tau ≥ 400 (2011–2016) or ≥ 200 (2008–2010) pg/mL.

### 2.4. PEA Testing

Inflammatory proteins panel testing was performed in 2017 using the multiplex PEA technology as previously described by Olink (Uppsala, Sweden) [10]. All the samples were run on the inflammation panel, which consists of 92 biomarkers (Table S1), with up to 96 samples tested simultaneously on each run. The Olink panel validation data are freely available online (https://www.olink.com/data-you-can-trust/validation/, accessed on 23 July 2021). The resulting data for each biomarker were expressed as normalized protein expression (NPX) value. NPX is an arbitrary unit on a log2 scale that is obtained by normalizing the concentration values to minimize inter- and intra-assay variations. A high NPX value corresponds to a high protein concentration and can be linearized by using the formula 2^NPX^. NPX values were subsequently z-scored to allow for a better comparison in multivariate analysis.

### 2.5. Statistical Analysis

Data analysis was performed by using the R software v 3.6 and *OriginPro* v 9.0. Continuous variables were reported as the mean ± standard deviation. The Kolmogorov-Smirnov (KS) normality test was used to disproof data normality. The significance of age and gender differences between OND and AD-MCI groups was assessed by means of the Mann–Whitney U-test and Fisher’s Exact test for count data, respectively. Receiver Operating Characteristic (ROC) curve analysis was performed by using the R package *pROC* [15]. The area under the curve (AUC) and AUC sensitivity and specificity confidence intervals were calculated by using 2000 bootstrap replicates. Age-adjusted AUC were instead computed with the R package *AROC*, according to the semiparametric Bayesian approach described by F. Machado e Costa and A. C. Braga [16]. The significance of z-score differences between groups was assessed by two-way analysis of covariance (ANCOVA) by assuming age as a covariate. Multiple testing effects were taken into account by correcting the significance level according to Bonferroni [17]. Considering that 46 different proteins were taken into account for the analysis (proteins with a call-rate of > 95%) and a significance level of 0.05, the Bonferroni correction would require changing the significance level to 0.0011. The correlation among z-scored proteins percentages was represented in terms of Spearman’s correlation coefficients and displayed in a heatmap by using the *pheatmap* R package [18]. Proteins were grouped according to a hierarchical clustering [19,20]. Correlation coefficients and Ward’s linkage [21] were used as distance and linkage parameters for clustering. Least absolute shrinkage and selection operator (LASSO) [22,23], a feature selection technique used for machine learning predictive modeling, was used to identify the biomarker panel that offered the best discriminatory performance. The *glmnet* R package was used for this purpose [24]. Within the AD-MCI group, correlations between the z-scores of the most discriminatory proteins and classical AD biomarkers were evaluated in terms of Spearman’s correlation coefficients.

## 3. Results

### 3.1. Characteristics of the Patients

A total of 34 AD-MCI patients (M/F 14/20, mean age 72.2 ± 5.8 y) were recruited for the study. None of the patients were taking any CNS-active drugs, such as acetylcholinesterase inhibitors or NMDA receptor modulators, at the time of diagnostic work-up. All patients underwent a clinical follow-up at least 1 year (mean follow-up 2.4 ± 1.3 y) after the baseline assessment. Baseline MMSE was 22.4 ± 4.1, while follow-up MMSE was 19.2 ± 5.7.

As neurological controls, a total of 25 subjects (M/F 15/10, mean age 66.8 ± 7.5 y) who underwent lumbar puncture for diagnostic purposes were enrolled. This group included patients with headache (*n* = 13), psychiatric disorders (*n* = 10), or mononeuropathy (*n* = 2). Subjects did not show significant differences in terms of gender distribution among the groups (*p* = 0.19, Fisher’s exact test), while the age of AD-MCI patients was significantly higher than those of OND subjects (*p* = 0.005, Mann–Whitney U-test).

### 3.2. PEA Testing

Out of the 92 proteins determined through the PEA technique, 46 had a call rate <95% (<95% of the participants had a valid measurement of that protein) and were removed from the analysis (Table S1).

### 3.3. Univariate Diagnostic Performance of PEA-Tested Proteins

We performed a univariate ROC analysis of the z-scores relative to the measured proteins (for abbreviations see Table S1). The AUC and age-adjusted *p*-values (ANCOVA) of the proteins that were most able to differentiate between AD-MCI and OND are shown in Table 1. Only proteins whose AUC was different from 0.5 within its 99.9% CI (*p* < 0.001) were considered, in order to take into account possible multiple testing effects. All the z-scores relative to the proteins presented in Table 1 were approximately normally distributed by the KS test. Thus, considering the significant age difference between the OND and AD-MCI groups, we also applied a two-way ANCOVA to assess the significance of the differences in z-scores between OND and AD-MCI by controlling for age. Notably, SIRT2 and HGF z-scores significantly differed between AD-MCI and OND with a *p* < 0.001, even after adjusting for age differences.

### 3.4. Correlation Analysis of PEA-Tested Proteins

**Figure 1:**
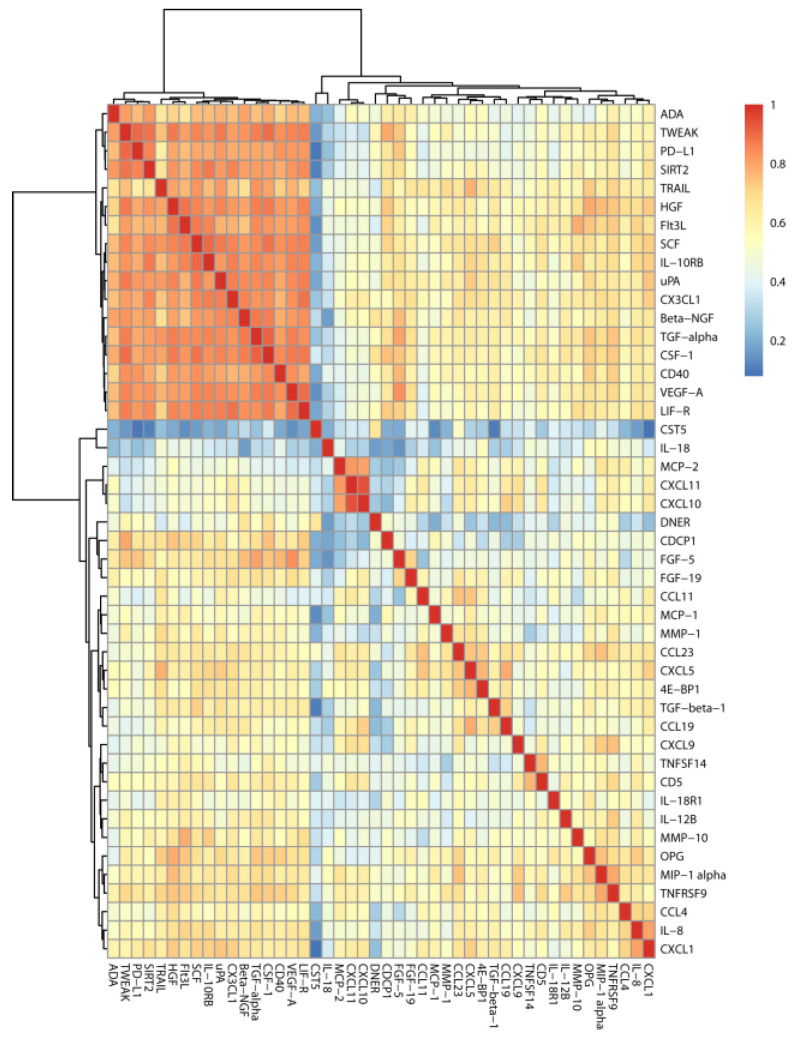
Correlation heatmap. Correlation coefficients were computed according to Spearman. Hierarchical clustering was used for ordering proteins by using correlation coefficients as distance and the Ward’s linkage criterion [21]. From the correlation and cluster analysis summarized in Figure 1, it emerges that, among the measured proteins, some of them strongly correlated with each other. The major cluster consisted in ADA, TWEAK, PD-L1, SIRT2, TRAIL, HGF, Flt3L, SCF, IL-10RB, uPA, CX3CL1, Beta-NGF, TGF-alpha, CSF-1, CD40, VEGF-A, and LIF-R. Another independent secondary cluster of highly correlated proteins consisted of MCP-2, CXCL11, and CXCL10. For abbreviations of PEA-tested proteins, see Table S1.

The majority of the z-scored NPX values in the OND and AD-MCI groups were approximately normally distributed by applying the KS test. However, since normality could be excluded for some analytes, we decided to quantify correlations among them in the entire cohort (AD-MCI and OND) with Spearman’s correlation coefficients. Only positive correlations were observed among the measured proteins in the OND and AD-MCI groups. By treating correlation coefficients as distances, we grouped together highly correlating proteins by hierarchical clustering. The result of this procedure is summarized in the heatmap shown in Figure 1.

### 3.5. Multivariate Diagnostic Performance of PEA-Tested Proteins

**Figure 2:**
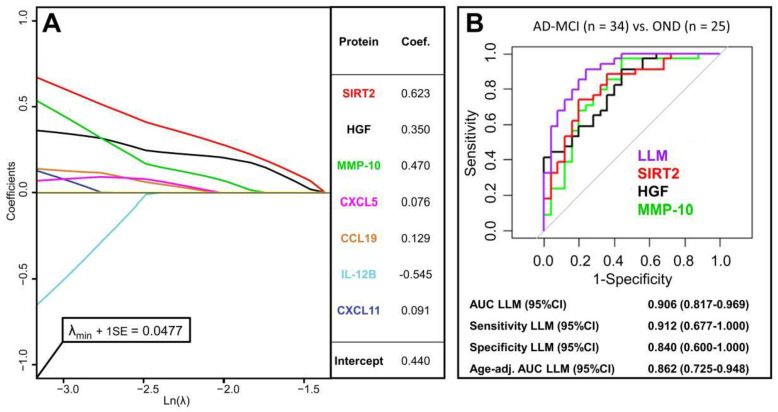
(**A**) LASSO coefficients in function of the shrinkage parameter λ from λ = 0.25924 (at least 1 coefficient ≠ 0) to λ = 0.0477 (λ_min_ + 1SE). The coefficients found for λ_min_ + 1SE were used to build a LASSO-based logistic model (LLM). (**B**) ROC curves relative to the diagnostic performance of LLM and the three proteins z-scores most contributing to the model, namely SIRT2, HGF, and MMP-10. For abbreviations of PEA-tested proteins, see Table S1.

Considering that some of the z-scores relative to the proteins shown in Table 1 were highly correlated, we decided to apply the LASSO method to identify a panel of proteins able to differentiate AD-MCI from OND while keeping the lowest possible dimensionality by eliminating the highest possible number of parameters among highly correlated variables. Being λ_min_, the shrinkage parameter corresponding to minimum binomial deviance between the multinomial logistics regression model and groups categories (optimal model), we selected the one at 1 standard error (λ_min_ + 1SE) from it, to select the simplest model with accuracy comparable to the optimal model, to avoid overfitting. The LASSO coefficients behavior from λ = 0 to λ = λ_min_ + 1SE, together with the diagnostic performance of the LASSO logistic model (LLM) and of the three main contributing proteins are shown in Figure 2. The linear combination of the selected proteins with the coefficients displayed in Figure 2A was significantly different between AD-MCI and OND (ANCOVA age-adjusted *p*-value = 2.97 × 10^−7^), and LLM differentiated AD-MCI from OND with an AUC of 0.906. Considering the age bias between AD-MCI and OND groups, age-adjusted AUC for LLM was also calculated and displayed in Figure 2B.

Moreover, repeating the procedure on a smaller age-matched cohort (ANOVA *p*-value = 0.39, U-test *p*-value = 0.15), by removing the 6 youngest OND subjects (age from 55 y to 60 y), produced a 6-variable model (SIRT2, coef. 0.655; CXCL5, coef. 0.465; HGF, coef. 0.102; IL-12B, coef. −0.325; MMP-10 coef. 0.0237 and CDCP1 coef. 0.027). The model (λ = 0.0616) provided an AUC of 0.876 (0.757–0.966) in the cohort subset, similar to the age-adjusted AUC obtained for the LLM in the whole cohort (Figure 2B).

**Figure 3:**
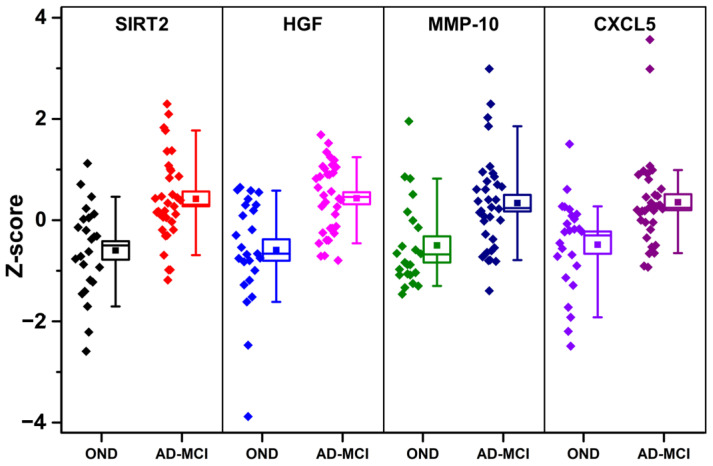
Distribution of SIRT2, HGF, MMP-10, and CXCL5 z-scores in OND and AD-MCI groups. Boxes representing data distributions are centered on the mean values, with the internal horizontal line representing the median. Box heights are equal to the standard error of mean values, whiskers represent the 10–90% data range. For abbreviations of PEA-tested proteins, see Table S1.

Among the tested proteins, SIRT2, HGF, MMP-10, and CXCL5 were present both in the LASSO selections and among the proteins showing the best performance in differentiating AD-MCI from OND (Table 1). The distributions of z-scored values relative to these proteins are shown in Figure 3.

### 3.6. Correlation Analysis between PEA-Tested Proteins and CSF AD Biomarkers

Within the AD-MCI group, correlation analysis between the z-scores of the most discriminatory PEA-tested proteins (listed in Table 1) and classical CSF AD biomarkers showed different significant associations (Table 2). A significant negative correlation was found between CSF Aβ42 and MMP-10 (ρ = −0.37, *p* < 0.05), while significant positive correlations were found between CSF p-tau and SIRT-2, HGF, uPA, LIF-R, and TWEAK, the most significant being those with SIRT2 (ρ = 0.48, *p* < 0.005), LIF-R (ρ = 0.51, *p* < 0.005), and TWEAK (ρ = 0.43, *p* < 0.005). Finally, a positive correlation was found between CSF t-tau and LIF-R (ρ = 0.35, *p* < 0.05).

## 4. Discussion

Our findings confirm the potential of the application of high-throughput multiplex assays in investigating novel pathophysiological pathways in neurodegenerative diseases such as AD. Although a study on an even broader panel of potential novel CSF AD biomarkers in a larger population has been recently performed [9], it was based on a multiplex mass spectrometry method that requires a large volume of CSF. An antibody-based method such as PEA may be more accessible and more easily translated into a clinical setting. Also, the population study included the entire AD continuum, while we focused only on AD-MCI patients, which makes our findings more specific to the prodromal AD stage. CSF multiplex PEA testing, coupled with a machine-learning statistical approach, led us to identify interesting biological pathways other than amyloidosis and tauopathy, potentially linked with AD pathophysiology in the early clinical phases of the disease (AD-MCI). This choice allowed us to better investigate the contribution of neuroinflammation as a potential early mechanism in AD, rather than as a consequence of advanced neurodegeneration. Moreover, the selection of patients with a CSF profile suggestive of AD (A+/T+) for a proteomics study ensured us a high level of biological homogeneity, thus reducing potential sources of variability.

In this specific cohort of early AD individuals, the subset of proteins that were present in the selected panel able to differentiate in single AD-MCI from controls with the highest accuracy were SIRT2, HGF, MMP-10, and CXCL5.

SIRT2 (sirtuin 2) is a protein deacetylase highly expressed in the mammalian CNS, mainly found in the cytoplasm of oligodendrocytes [25]. It is particularly expressed in the cortex, striatum, hippocampus, and spinal cord, but its functions are still largely unknown [25]. SIRT2 has demonstrated a possible role in mitosis regulation, genome integrity, cell differentiation, cell homeostasis, but also in microglial activation, and hampering autophagic activity [25]. Interestingly, SIRT2 mRNA expression has been found to be increased in the peripheral blood of AD patients, and an association between SIRT2 polymorphisms and AD risk has been identified [26,27]. Inhibition of SIRT2 has demonstrated effects in counteracting the amyloidogenic pathway in vitro, by reducing Aβ production as well as its toxic effects on neurons [28,29]. Evidence also suggests that SIRT2 inhibition may induce α-tubulin acetylation thus decreasing tau-phosphorylation [30]. Interestingly, from our analysis, CSF SIRT2 positively correlated with CSF p-tau levels (ρ = 0.48, *p* < 0.005) in AD-MCI patients, thus supporting the evidence of a possible association between SIRT2 activity and tau pathology. Moreover, since we found higher levels of SIRT2 in the CSF of AD-MCI patients compared to controls, it is possible to hypothesize that an overexpression of this protein may be associated with AD pathophysiology in the earliest phases of the disease.

HGF (hepatocyte growth factor), a potent mitogen for hepatocytes, is expressed, together with its high-affinity tyrosine kinase receptor (Met), in the CNS, and specifically in the hippocampal region [31]. It has been demonstrated that synaptic activity modulates HGF signaling in hippocampal neurons and, in turn, HGF modulates synaptic function and enhances dendritic arborization [32,33], suggesting a potential physiological role of HGF signaling in synaptic functions. Of interest, in AD brains, higher staining for HGF in astrocytes within the parietal and temporal lobe white matter compared to controls has been documented [34]. Moreover, in CSF, a higher concentration of HGF in AD patients vs. controls has already been found by means of ELISA measurement [35]. It has been hypothesized that the higher values of CSF HGF may depend upon the activation of astrocytes or an increased number of astrocytes in the white matter in AD [35], therefore serving as a biomarker of glial activation in neurodegeneration. According to our findings, such processes are therefore already evident and quantifiable in the CSF in the earliest prodromal phases of the disease, with a positive correlation with markers of tau pathology. The latter finding suggests a potential pathophysiological association between tau hyperphosphorylation and astrocytes activation as measured with CSF HGF.

MMP-10 (matrix metalloproteinase-10 or stromelysin-2) belongs to the family of matrix metalloproteinases, which are enzymes able to degrade components of the extracellular matrix. In the CNS, they are expressed in neurons but are also secreted by astrocytes and microglia [36,37,38]. Of interest, matrix metalloproteinases are able to degrade Aβ in vitro as well as in vivo in animal models of cerebral amyloidosis [39,40]. By means of multiplex bead-based immunoassays, MMP-10 has already been found to be increased in the CSF of AD patients compared to controls, as was its CSF/plasma ratio compared to other forms of dementia (namely, vascular dementia) [41]. Additionally, MMP-10 was found to be increased in the CSF of AD-dementia patients and patients with MCI and a CSF A+ profile compared to controls in a previous study based on the same multiplex testing and on a similar statistical approach [42]. The concordance of our findings, though obtained on a different population study, supports the possibility of MMP-10 as a biomarker of AD. Since MMP-10 is known to be highly expressed by activated microglia [43], it may be involved in the non-neuronal cells’ response to neuronal damage, thus playing an important pathophysiological role in neurodegeneration. Also, in AD patients, a positive correlation between CSF MMP-10 and both total tau and phosphorylated tau has been documented [41]. Of interest, in our work, we did not find significant correlations between CSF MMP-10 and p-tau/t-tau, while MMP-10 protein levels were found to be negatively correlated with CSF Aβ42 levels in AD-MCI patients (ρ = −0.37, *p* < 0.05), thus supporting a possible interaction between this specific metalloproteinase and amyloid pathology in AD patients.

CXCL5 (C-X-C motif chemokine 5) is a small cytokine that is produced by immune and vascular endothelial cells in response to proinflammatory cytokines through NF-kB activation [44]. Its CSF levels have been found to be increased in CNS inflammatory diseases, such as multiple sclerosis, being involved in blood-brain barrier (BBB) dysfunction [45]. Previously, a CSF increased concentration of CXCL5 in AD patients has not been described. Therefore, our findings support a possible role of immune mediators involved in BBB stability even in neurodegenerative diseases such as AD.

Of interest, the correlation and cluster analysis of all the CSF markers we measured with PEA showed at least two clusters of highly correlated protein levels in the entire study cohort (i.e., both AD-MCI and OND). The major cluster included SIRT2 and HGF, together with another 15 markers (Figure 1), suggesting that the neuroinflammatory mechanisms linked to AD involve multiple biological actors, among which SIRT2 and HGF were the most altered in the AD-MCI group with respect to OND.

Another independent secondary cluster included a small group of cytokines (MCP-2, CXCL11, and CXCL10) that are chemotactic for and activate many different immune cells that are involved in inflammatory response.

Taken together, our findings shed light on the potential contribution of immune-mediated mechanisms in the pathophysiology of AD. SIRT2 overexpression, together with astrocytic and microglial activation and BBB dysfunction seem to be the most promising mechanisms that can be quantified in the CSF of early-stage AD patients. As a limitation of our study, we must consider the limited sample size. Further replication data in larger cohorts are therefore needed.

## 5. Conclusions

- We present promising findings using PEA biomarker technology coupled with a machine-learning statistical approach for the discovery of novel markers and pathophysiological mechanisms of AD.
- Our findings suggest an association between AD pathology and the SIRT2 pathway, astrocyte and microglia activation, and BBB dysfunction, as reflected by concentration changes in the CSF of SIRT2, HGF, MMP10, and CXCL5.
- Studies confirming these results in larger cohorts also by means of different assays, such as the single-molecule array and ELISA, might be needed to validate PEA as a promising tool for biomarker discovery in the field of neurological diseases.

## Figures and Tables

**Table 1:** List of most discriminatory proteins between AD-MCI and OND by univariate analysis.

Protein Name	AUC ^1^	*p*-Value ^2^
SIRT2	0.809	5.2 × 10^−4^
HGF	0.802	6.4 × 10^−4^
MMP-10	0.793	3.2 × 10^−3^
pIL-10RB	0.786	1.3 × 10^−3^
uPA	0.771	4.0 × 10^−3^
CXCL5	0.761	2.3 × 10^−3^
LIF-R	0.760	3.5 × 10^−3^
CX3CL1	0.757	6.2 × 10^−3^
SCF	0.752	3.4 × 10^−3^
Flt3L	0.752	4.0 × 10^−3^
TWEAK	0.747	7.7 × 10^−3^

^1^ Greater than 0.5 within 99.9% CI. ^2^ From the ANCOVA. Legend. AUC: area under the curve. For abbreviations of PEA-tested proteins, see Table S1.

**Table 2:** Spearman’s correlation coefficients between the most discriminatory PEA-tested proteins and classical AD biomarkers within the AD-MCI group. For abbreviations of PEA-tested proteins, see Table S1.

Protein Name	Aβ42	p-tau	t-tau
SIRT2	0.07	0.48 **	0.33
HGF	−0.17	0.37 *	0.35
MMP-10	−0.37 *	0.20	0.09
IL-10RB	−0.11	0.29	0.17
uPA	0.00	0.37 *	0.29
CXCL5	−0.17	−0.02	0.07
LIF-R	−0.03	0.51 **	0.35 *
CX3CL1	0.01	0.42 *	0.30
SCF	−0.03	0.25	0.17
Flt3L	−0.14	0.25	0.17
TWEAK	−0.09	0.43 **	0.29

* 0.01 < *p* < 0.05; ** 0.001 < *p* < 0.005.

## Data Availability

The data presented in this study are available on request from the corresponding author upon reasonable request. The data are not publicly available due to privacy and ethical reasons.

## Supplementary Materials

The following are available online at https://www.mdpi.com/article/10.3390/cells10081930/s1, Table S1: List of the biomarkers measured by means of PEA technique (inflammation panel) in the CSF of recruited patients.
